# Ab initio phase diagram and nucleation of gallium

**DOI:** 10.1038/s41467-020-16372-9

**Published:** 2020-05-27

**Authors:** Haiyang Niu, Luigi Bonati, Pablo M. Piaggi, Michele Parrinello

**Affiliations:** 1 State Key Laboratory of Solidification Processing, International Center for Materials Discovery, School of Materials Science and Engineering, Northwestern Polytechnical University, Xi’an, 710072 P. R. China; 2 Department of Chemistry and Applied Biosciences, ETH Zurich c/o USI Campus, Via Giuseppe Buffi 13, 6900 Lugano, Switzerland; 3 Facoltà di Informatica, Instituto di Scienze Computationali, and National Center for Computational Design and Discovery of Novel Materials MARVEL, Università della Svizzera italiana (USI), Via Giuseppe Buffi 13, 6900 Lugano, Switzerland; 4 Department of Physics, ETH Zurich, c/o Università della Svizzera italiana, Via Giuseppe Buffi 13, CH-6900 Lugano, Switzerland; 5 Istituto Italiano di Tecnologia, Via Morego 30, 16163 Genova, Italy

**Keywords:** Phase transitions and critical phenomena, Structure of solids and liquids, Computational methods

## Abstract

Elemental gallium possesses several intriguing properties, such as a low melting point, a density anomaly and an electronic structure in which covalent and metallic features coexist. In order to simulate this complex system, we construct an ab initio quality interaction potential by training a neural network on a set of density functional theory calculations performed on configurations generated in multithermal–multibaric simulations. Here we show that the relative equilibrium between liquid gallium, *α*-Ga, *β*-Ga, and Ga-II is well described. The resulting phase diagram is in agreement with the experimental findings. The local structure of liquid gallium and its nucleation into *α*-Ga and *β*-Ga are studied. We find that the formation of metastable *β*-Ga is kinetically favored over the thermodinamically stable *α*-Ga. Finally, we provide insight into the experimental observations of extreme undercooling of liquid Ga.

## Abstract (web summary)

Exploring nucleation processes of gallium by molecular simulation is extremely challenging due to its structural complexity. Here the authors demonstrate a neural network potential trained on a multithermal–multibaric DFT data for the study of the phase diagram of gallium in a wide temperature and pressure range.

## Introduction

**Fig. 1:**
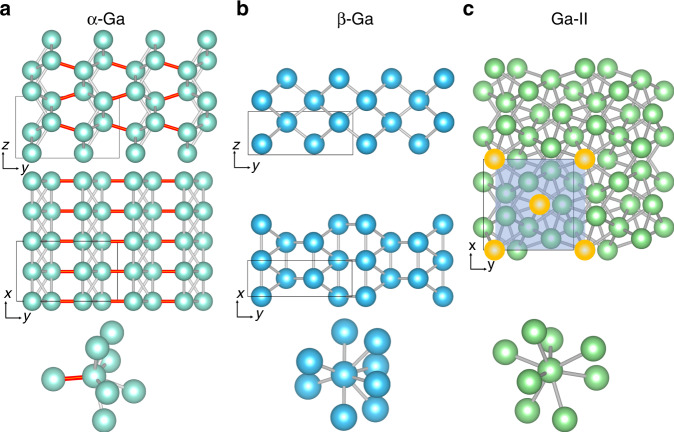
Crystal structures of solid gallium phases. **a** *α*-Ga, **b** *β*-Ga, and **c** Ga-II phases including the atomic nearest neighbor environments of each phase. The covalent bond of the Ga_2_ dimer in *α*-Ga is highlighted with red color. Ga-II can be described as interpenetrated BCC lattices and one of them is highlighted by yellow atoms with a blue-shaded area.

Elemental gallium is a unique metal with a number of fascinating and unusual properties^1–6^. In its different phases it finds a variety of important technological applications^1,7^. Unlike most metals, including those in the same column of the periodic table, it crystallizes in rather complex structures^5,8^ as illustrated in Fig. 1. Its stable solid phase at ambient conditions, known as *α*-Ga, is orthorhombic with four atoms in the primitive unit cell^8^. Each atom is coordinated to seven neighbors, resulting into a highly anisotropic atomic environment^3^ (see Fig. 1a). The bonding between the two nearest neighbor atoms is described as covalent and *α*-Ga is thought to have a mixture of covalent and metallic bonds.

Upon melting, like water, it exhibits a density anomaly with the liquid expanding as much as 3.1%. Despite decades of experimental investigations, the structure of liquid gallium is not yet fully understood^2,6^. Furthermore, micrometer-sized or submicrometer-size liquid gallium could be undercooled down to 150 K without solidification^4,9–11^. In such scenario, the crystallization does not produce the stable *α* phase but mostly the *β*-Ga structure. Experiments also showed that Ga nanoparticles in the range of 3–15 nm can be undercooled even down to 90 K with no sign of a freezing transition^12^. The structure of *β*-Ga is monoclinic and contains square-like arrangements of atoms forming layers parallel to the (021) plane as shown in Fig. 1b. At variance with *α*-Ga, each atom in *β*-Ga has eight nearest neighbors at the distance of 2.78 *Å*. In addition the Ga phase diagram exhibits a variety of different complex stable and metastable polymorphs, such as the *δ*-Ga, *γ*-Ga, and Ga-II phases^4,5^. Among them, Ga-II shows thermodynamic stability at high pressure and can be described as interpenetrating body-centered-cubic lattices with each atom having eightfold coordination (Fig. 1c). The structural complexity of liquid and solid Ga makes the study of nucleation and relative equilibria using molecular simulations very challenging.

In order to perform the simulations, two hurdles need to be cleared. One is the long-time scale over which nucleation takes place, whereas the other is an accurate description of the subtle interaction that leads to the complex behavior of Ga. Luckily, much progress has been made in both areas. On the one hand, efficient enhanced sampling methods have allowed studying crystallization in a variety of systems^13–17^. As far as describing accurately the Ga interaction is concerned, an ab initio description is called for but running first-principles molecular dynamics (MD) is prohibitively expensive. The solution to this conundrum has been first suggested by Behler and Parrinello^18^ and consists in training a neural network (NN) on a large number of appropriately selected set of configurations. This implies calculating accurate total energies and forces, typically at the density functional theory (DFT) level and optimize the parameters of a NN so as to best reproduce these data. The ability of NNs to represent functions of many variables in a continuous way and to interpolate within the training set, allows to obtain a faithful representation of the ab initio potential energies and forces at a much reduced cost. The work of Behler and Parrinello^18^ has been developed and applied in many areas. If we confine ourselves to condensed matter applications one can quote refs. ^19–21^ as well as the work of Bartok et al., which uses Gaussian processes rather than NNs to represent the potential^22^. Here, we choose to use the recently developed deep potential for MD (DeePMD) method^21,23^. We find that the obtained NN force field can describe the structural and other related properties of *α*-Ga, *β*-Ga, Ga-II, and liquid gallium well. Importantly, the phase diagram of gallium in a wide temperature and pressure range are calculated, which shows in good agreement with experimental results. By comparing the nucleation properties of *α*-Ga and *β*-Ga, we find that the formation of metastable *β*-Ga is kinetically favored over the thermodynamically stable *α*-Ga above 174 K.

## Results

### NN force field training

**Fig. 2:**
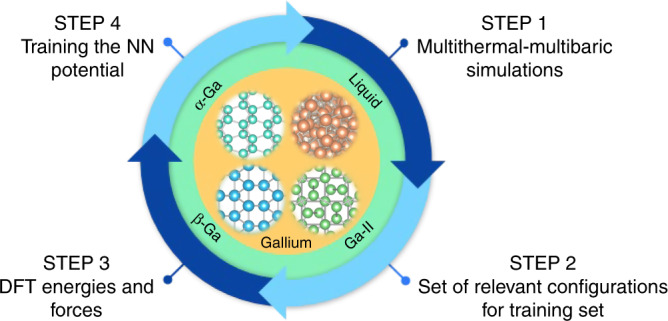
The neural network training procedure. Four steps, i.e. mutithermal-multibaric simulations, set of relevant configurations collection, energies and forces calculation at the DFT level, and the NN potential training, are involved in the iterating procedure.

A careful choice of the configurations used in the training of the NN^17,24^ is crucial to the success of the NN potential training procedure. As our focus is on the study of the Ga phase diagram and its nucleation process, we need all the characteristic structures of the liquid, the solid, and most importantly, of the nucleation region in which the two phases coexist. Obtaining all the relevant configurations is far from trivial, notably the ones that are far from the equilibrium. To address this issue, we take inspiration from the work by Bonati and Parrinello^17^ in which the training configurations were collected from a metadynamics^25,26^ run that used a standard empirical potential to study the liquid–solid phase transition. In the present case, as we set out to study a wide region of the Ga phase diagram, we generalize the procedure of Bonati and Parrinello^17^, and we train our NN on simulations performed in the multithermal–multibaric ensemble^27,28^ so as to present the NN with all the relevant configurations. The NN training procedure includes four steps as illustrated in Fig. 2. These steps are the multithermal–multibaric simulation^27^, the collection of a set of relevant configurations for the training set, the calculation the energies and forces at the DFT level, and training the NN potential. This procedure is based on the combination of three methods, namely the multithermal–multibaric simulation, the choice of efficient collective variables (CVs)^28^ and the use of the DeePMD method. By iterating this procedure, we are able to obtain a DFT quality potential.

**Fig. 3:**
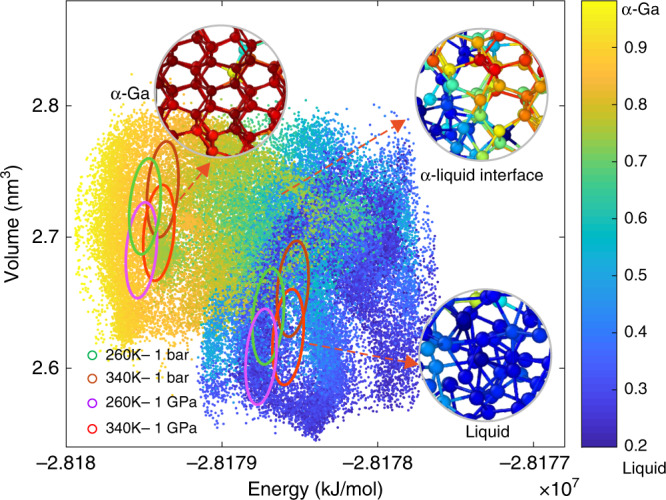
Energy-volume regions explored with different ensembles. The dots correspond to the region sampled in the multithermal–multibaric ensemble for the nucleation of *α*-Ga and are colored according the collective variable defined in Eq. (13). The higher the value of the collective variable, the more solid-like a configuration is. The ellipses show the regions sampled in the isothermal–isobaric ensemble both for *α*-Ga and liquid at four different conditions, 260 K - 1 bar, 340 K - 1 bar, 260 K - 1 GPa and 340 K - 1 GPa. Inset: structure of *α*-Ga, *α*-liquid interface, and liquid gallium.

It is worth noting that the use of multithermal–multibaric simulations is a key feature of our approach, which allows us to build a reliable NN potential and, in turn, to determine the Ga phase diagram. In such simulations, we explore a large variety of arrangements, from those typical of liquid Ga to the *α*-Ga ones passing through intermediate states. As we report in Fig. 3, these configurations cover a much wider range of energies and volumes compared with standard isothermal–isobaric simulations. Exposing the NN to such a variety of physically relevant configurations is key to training an NN potential valid in a whole-temperature pressure region. Furthermore, employing such simulations allows defining clearly the range of validity where the NN potential can be trusted. This is very important in the context of NNs, as we have no exact way to estimate their ability to generalize.

### Structure of liquid gallium

**Fig. 4:**
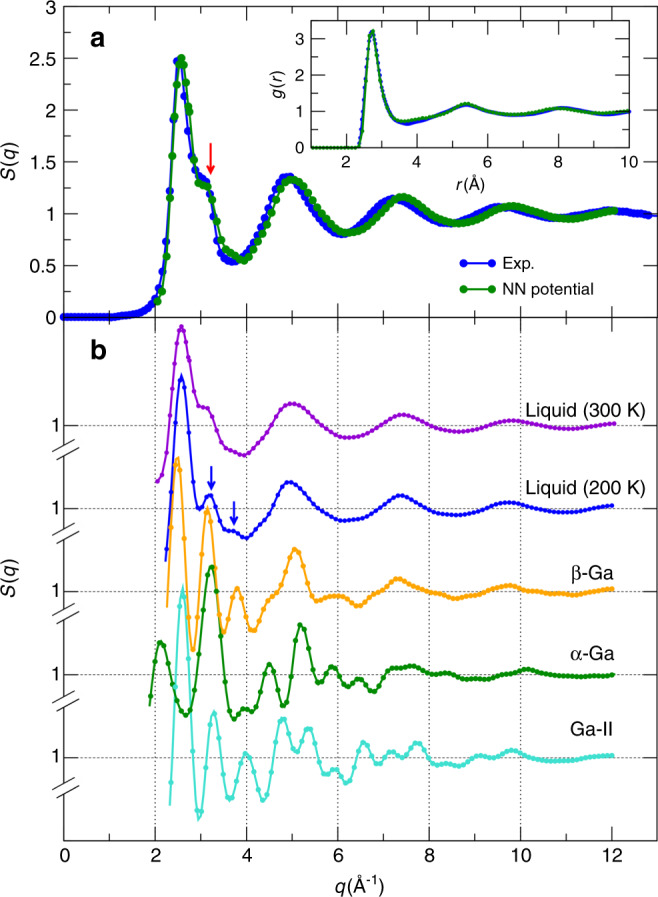
Analysis of the structure of liquid gallium. **a** Calculated static structure factor *S*(*q*) of liquid gallium at 300 K and 1 bar, and comparison with experimental results taken from ref. ^2^. Inset: radial distribution function *g*(*r*) of liquid gallium. **b** Calculated structure factor *S*(*q*) of liquid gallium at different temperatures and 1 bar. Structure factors *S*(*q*) of *α*-Ga, *β*-Ga, and Ga-II are also shown here to highlight the structural similarity between the liquid and *β*-Ga.

Once the NN potential is obtained, we can use it to study the structure of liquid gallium and its nucleation into different phases. The radial distribution function *g*(*r*) and the static structure factor *S*(*q*) of liquid gallium obtained with the NN force field are shown in Fig. 4, which are in accordance with the corresponding experimental results. By integrating the *g*(*r*) of liquid Ga out to the first minimum, one gets an average coordination number of 11.5, which is similar with liquid metals with close-packed structure, indicating that its atomic environment has a higher degree of isotropy than *α*-Ga. These features could be expected to contribute to the density anomaly, that results in an expansion of ~3.1% upon freezing into *α*-Ga. Furthermore, a shoulder on the high-*q* side of the *S*(*q*) is observed, in good agreement with experimental observations. We have also compared the *S*(*q*) of the liquid with that of the *α*, *β,* and Ga-II phases. The results show that the *S*(*q*) of *β*-Ga exhibits a fair agreement with the positions of the peaks of the liquid gallium *S*(*q*) as shown in Fig. 4b, indicating the underlying structural similarity between *β*-Ga and the liquid. On the other hand, significant discrepancy could be observed between *α*-Ga and liquid gallium, in agreement with results reported in previous work^6^. Even though the *S*(*q*) of Ga-II also shows some similarity with that of liquid gallium, the role of Ga-II is not important in this case since at ambient temperature and pressure Ga-II is highly unstable^5^. We have also calculated the *g*(*r*) and the *S*(*q*) of liquid gallium for different temperatures and pressures (see Supplementary Fig. 1). The results show that liquid gallium becomes more structured as the temperature decreases and pressure increases.

### Ab initio phase diagram

**Fig. 5:**
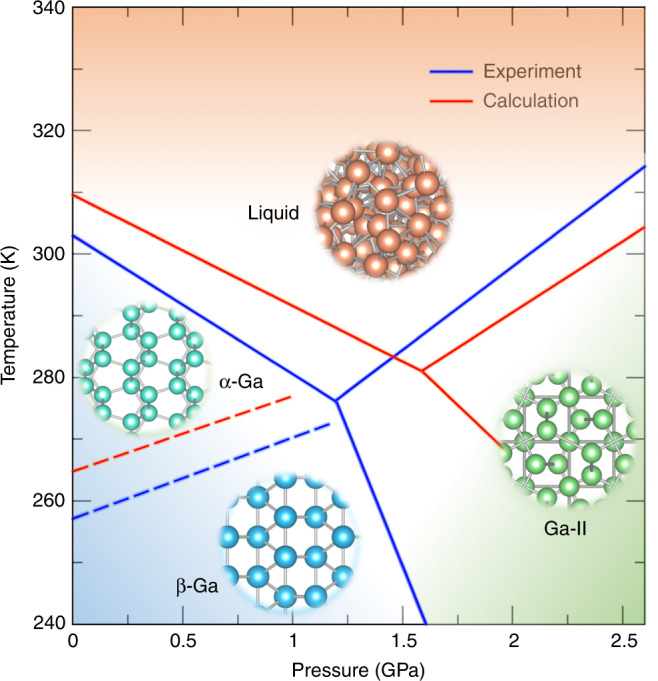
Phase diagram of gallium. The calculated results from the multithermal–multibaric simulations and the experimental results are colored with red and blue lines, respectively. The coexistence lines between thermodynamically stable phases are shown in solid lines, whereas the coexistence line between metastable *β*-Ga and the liquid is shown with a dashed line. Inset: Structures of *α*-Ga, *β*-Ga, Ga-II, and liquid gallium.

Now we turn our attention to the solid phases of Ga and their relative equilibrium with liquid gallium. Here, we aim at calculating the phase diagram in the range 240–340 K and 0–2.6 GPa. To achieve this result, we have performed three multithermal–multibaric simulations. These simulations were designed to explore the reversible transitions between the liquid and the different crystal structures (see Supplementary Fig. 2). Then we followed the procedure discussed in ref. ^28^ to calculate the free energy difference between the liquid and the solid phase Δ*G*_*l*→*s*_(*T*, *P*) as a function of temperature and pressure (see Supplementary Fig. 3). The coexistence lines of the phase diagram could be obtained equating the free energies in the two states *G*_*l*→*s*_(*T*, *P*) = 0. It should be noted here that the coexistence line between *α*-Ga and Ga-II is estimated through *G*_*l*→*α*_(*T*, *P*) − *G*_*l*→*II*_(*T*, *P*) = *G*_*α*→*II*_(*T*, *P*) = 0. The phase diagram of gallium obtained from these three multithermal–multibaric simulations is shown in Fig. 5, and is in agreement with experimental data^5^. The triple point liquid-*α*-II is located at 280.9 K and 1.58 GPa, which is comparable with the experimental one of 276.2 K and 1.19 GPa^5^.

We have summarized in Table 1 the melting temperatures and lattice parameters of the solid phases investigated here. Both the melting temperatures and the lattice parameters are in good agreement with experiments. For instance, the melting temperature of *α*-Ga is estimated to be 309.5 ± 6 K, which is slightly higher than the experimental value 302.9 K.

**Table 1:** Melting temperature and lattice parameters of gallium phases.

Phase	*T*_*m*_ (K)	Lattice parameters (*Å* or ^∘^)	
*α*	302.9	*a* = 4.514; *b* = 7.644; *c* = 4.526	Exp.
	309.5 ± 6	*a* = 4.47 ± 0.02; *b* = 7.58 ± 0.04; *c* = 4.48 ± 0.02	Cal.
*β*	256.9	*a* = 2.766; *b* = 8.053; *c* = 3.332; *β* = 92.03	Exp.
	264.7 ± 5	*a* = 2.69 ± 0.02; *b* = 7.92 ± 0.04; *c* = 3.372 ± 0.02; *β* = 92.2 ± 0.5	Cal.
Ga-II	314.2	*a* = 5.951	Exp.
	304.3 ± 6	*a* = 5.89 ± 0.03	Cal.

The calculated (Cal.) and experimental (Exp.) melting temperatures *T*_*m*_ and lattice parameters of the α-Ga, β-Ga and Ga-II phases are listed. The theoretical and experimental data of Ga-II were obtained at 2.6 GPa, whereas the rest of the data were obtained at ambient pressure. The experimental results are taken from ref. ^5^.

### Nucleation of *α*-Ga and *β*-Ga

**Fig. 6:**
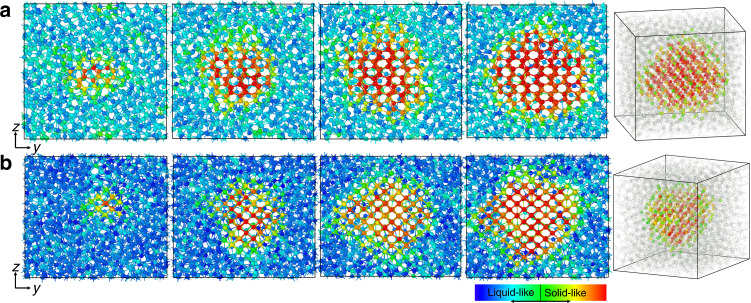
Snapshots from the nucleation process of gallium. **a** *α*-Ga at 280 K and 1 bar in a 2650 atoms system and **b** *β*-Ga at 240 K and 1 bar in a 2400 atoms system. The nuclei of the last snapshots are also shown in three-dimensional perspective and the liquid-like atoms are semi-transparent for clarity. Atoms are colored according to the descriptor defined in Eq. (12).

In order to investigate the nucleation behavior of the solid phases, we have performed several metadynamics simulations using the NN potential. Snapshots from the homogeneous nucleation process of *α*-Ga and *β*-Ga are illustrated in Fig. 6. Similar features could be observed in both cases. Initially, crystalline embryos form stochastically in the liquid phase. Eventually one cluster dominates and grows steadily into a large crystal. Interestingly, in the *y*–*z* plane the crystalline cluster of *α*-Ga appears relatively spherical, whereas the one of *β*-Ga is somewhat faceted and shaped as a parallelogram with rounded edges.

From a three-dimensional perspective, the nuclei in both cases are not far from a sphere as can be seen in Fig. 6. This is an important insight into the nucleation mechanism of Ga and will be used below to study this process within the framework of classical nucleation theory (CNT).

### Role of Ga_2_ dimers in the nucleation of *α*-Ga

**Fig. 7:**
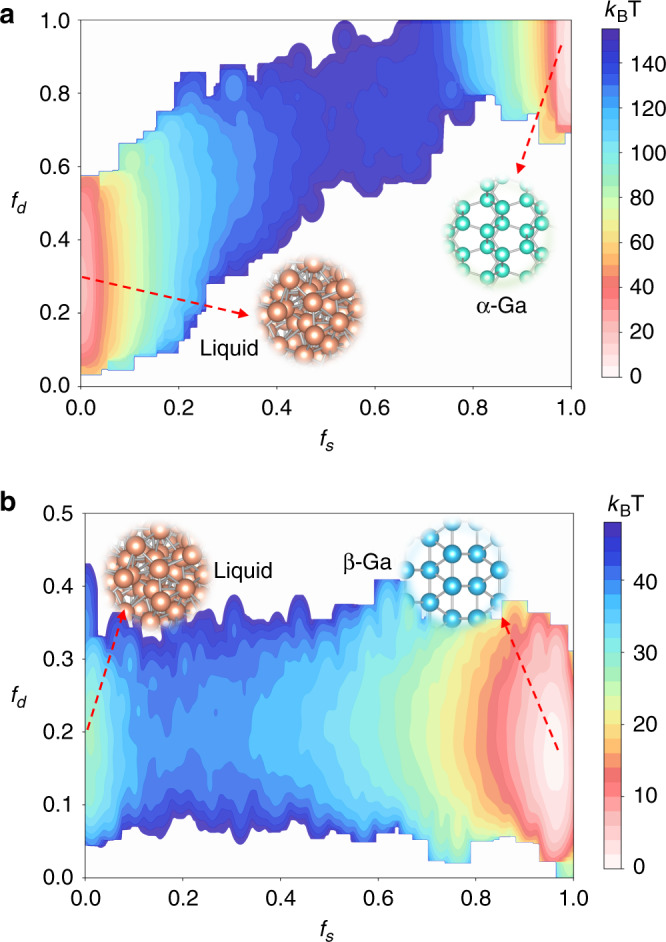
Reweighted free energy surfaces for the nucleation of gallium. **a** *α*-Ga at 280 K and 1 bar and **b** *β*-Ga at 240 K and 1 bar. The free energy surfaces are plotted as a function of the fraction of solid-like atoms *f*_*s*_ and fraction of Ga_2_ dimers *f*_*d*_.

Another question underlying the nucleation process of *α*-Ga is how the Ga_2_ dimers characteristic of this phase evolve. In order to address this issue, we have calculated the free energy surface (FES) as a function of the fraction of solid-like atoms *f*_*s*_ and fraction of Ga_2_ dimers *f*_*d*_ at 280 K (*T* ≈ 0.9 ${T}_{m}^{\alpha }$) and 1 bar with 144 Ga atoms in the system. It is worth mentioning that in order to get a converged FES, a much smaller system than the one reported in Fig. 6 is used here. As shown in Fig. 7a, the transition between *α*-Ga and liquid is well described by our CVs. In the solid phase, both the *f*_*s*_ and the *f*_*d*_ are equal to 1, wherein the liquid phase they are approximately equal to 0 and 0.25, respectively. The two basins are roughly linearly connected, indicating the strongly correlation between the Ga_2_ dimers and the formation of solid-like atoms. In the liquid state, the Ga_2_ dimers are randomly oriented, and the ones along the nucleation direction favors the formation of solid-like atoms. In contrast, the FES as a function of the fraction of solid-like atoms *f*_*s*_ and fraction of Ga_2_ dimers *f*_*d*_ of *β*-Ga nucleation at 240 K (*T* ≈  0.9 ${T}_{m}^{\beta }$) and 1 bar in the system with 144 atoms is shown in Fig. 7b. Owing to thermal fluctuation, a small fraction of dimers exists also in *β*-Ga. There is no marked change of *f*_*d*_ during the nucleation process of *β*-Ga owing to the structure similarity between liquid and *β*-Ga. These simulations shed light on the evolution of the Ga_2_ dimers in the nucleation of *α*-Ga. However, we could not quantitatively compare the nucleation barriers of *α*-Ga and *β*-Ga owing to severe finite size effects.

### Competition between *α*-Ga and *β*-Ga

In order to overcome the finite size effects and unravel the puzzling competition between *α*-Ga and *β*-Ga in undercooled liquid Ga, we have resorted to the seeding technique^29^. Under the assumption that CNT holds, this technique can be used to obtain the critical nucleus size and the nucleation energy barrier at moderate supercooling. Usually, a spherical cluster is inserted into the bulk liquid to create the initial simulation system. Here we improved on the standard procedure by using configurations extracted from the metadynamics simulations (Fig. 6) as starting points of the seeding simulations. The advantage of our strategy is that problems such as interface mismatching owing to the insertion of an arbitrary cluster can be avoided, as in our case the cluster is nucleated from the bulk liquid itself. Nevertheless, the configurations extracted from the nucleation of *α*-Ga and *β*-Ga were equilibrated for ~0.2 ns at the target temperature. We have chosen five initial configurations for each phase, and then we performed MD runs at different temperatures and monitored the cluster size to determine the temperature at which each cluster is critical.

As detailed in the methods section, in the CNT framework the nucleation energy barrier can be calculated as $\Delta G=\frac{1}{2}| \Delta \mu | {N}_{c}$, where *N*_*c*_ is the critical nucleus size and Δ*μ* the chemical potential difference between the liquid and solid phase. The latter can be approximated with the enthalpy change at melting as follows: $\Delta \mu =\Delta {H}_{m}(1-\frac{T}{{T}_{m}})$ (see ref. ^30^). The enthalpy of fusion *Δ*H_*m*_ of *α*-Ga is estimated to be 5.27 kJ/mol, in good agreement with the experimental value, 5.58 kJ/mol^31^.

**Fig. 8:**
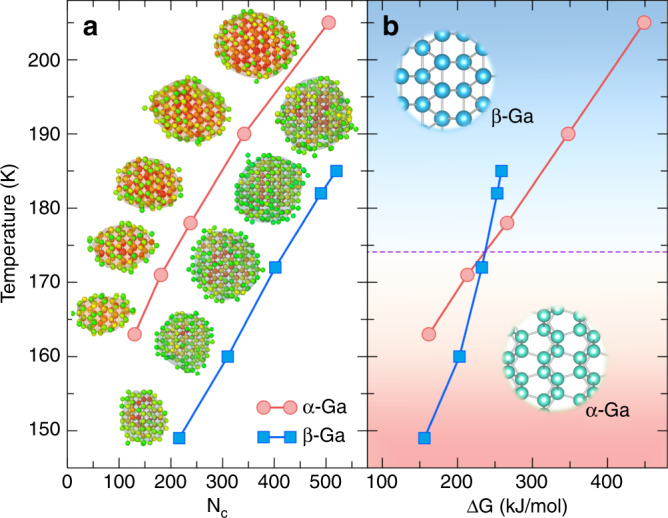
Nucleation properties of gallium. Temperature dependence of **a** the critical nucleus size N_*c*_ and **b** the nucleation energy barrier ΔG of *α*-Ga and *β*-Ga. The snapshots in **a** depict the critical nuclei at each temperature studied. The dashed line in **b** separates the figure into two zones, above the line *β*-Ga has a lower nucleation energy barrier than *α*-Ga and below the line the opposite is true. The uncertainties of N_*c*_ and ΔG are ~10 atoms and 8 kJ/mol, respectively.

In Fig. 8, we report the critical nucleus size *N*_*c*_ and the nucleation energy barrier for *α*-Ga and *β*-Ga at different temperatures.

First, one can see that the critical size of *β*-Ga is much larger than that of *α*-Ga at the same temperature. Furthermore, the curves of the nucleation barrier of the two crystalline phases intersect at a temperature of 174 ± 3 K. Above this temperature, *β*-Ga has lower nucleation energy barrier than *α*-Ga, which indicates that undercooling liquid Ga favors nucleation into *β*-Ga rather than *α*-Ga. Our results explain experimental findings that the crystallization of liquid Ga with micrometer size or submicrometer size does not form *α*-Ga but mostly *β*-Ga^4,9–11^. We also compare the interfacial free energy *γ* of these two cases exploiting the fact that *γ* ∝ ∣Δ*μ*∣N${}_{c}^{1/3}$ (see Eq. (16)). The results show that the interfacial energy of *β*-Ga is about three times smaller than that of *α*-Ga, this could be ascribed to the structural similarity between *β*-Ga and the liquid we reported earlier.

The nucleation rates *J* of *α*-Ga and *β*-Ga at 170 K and 180 K are estimated following Eq. (18) to be ~ 2.4 × 10^−23^ and 5.3 × 10^−31^ m^−3^ s^−1^, respectively. In other words, on average one would have to wait ~4.8 × 10^17^ and 2.2 × 10^25^ days to see a single nucleus forming in 1 m^3^ of liquid Ga at 170 K and 180 K, respectively. This suggests that homogeneous nucleation will not take place in these conditions and that heterogeneous nucleation is expected to be the normal crystallization mechanism, in accordance with experimental findings^11^. Our results indicate also why liquid Ga could be experimentally undercooled to extremely low temperatures before the onset of nucleation^4,9–11^.

## Discussion

In this work, we have combined a number of state-of-the-art computational techniques in order to construct a NN force field for gallium, which has many complex bonding and structural properties. The results show that the obtained NN force field can not only describe the structure of liquid gallium well, but it also captures the properties of the investigated solid phases *α*-Ga, *β*-Ga, and Ga-II. These properties include the melting temperatures, lattice parameters, and enthalpy of fusion. In addition, we have obtained the phase diagram of gallium in a wide temperature and pressure range, which is in good agreement with experimental findings. Considering the subtleties of the interactions involved, the agreement between theory and experiment is excellent. Furthermore, we have also studied the nucleation of *α*-Ga and *β*-Ga, and we show that above 174 K the formation of metastable *β*-Ga is kinetically favored over the thermodynamically stable *α*-Ga. The extremely high nucleation barriers for both *α*-Ga and *β*-Ga make the crystallization of liquid Ga very hard, which explains the experimental observations of extreme undercooling of liquid Ga. We believe our study thus offers a path to calculate phase diagrams and study the nucleation of complex materials with ab initio accuracy at an affordable cost.

## Methods

### Multithermal–multibaric simulation

The basic idea behind the variationally enhanced sampling (VES)^32^ based multithermal–multibaric simulation^27^ can be more simply grasped if we confine ourselves to a description of the multicanonical ensemble.

We recall that in the VES^32^ one introduces a functional of the bias *V*(*s*),

1 $$\Omega [V]=\frac{1}{\beta }{\mathrm{log}}\,\frac{\int\ d{\bf{s}} \, {e}^{-\beta \left[F({\bf{s}})+V({\bf{s}})\right]}}{\int\ d{\bf{s}} \, {e}^{-\beta F({\bf{s}})}}+\int\ d{\bf{s}} \, p({\bf{s}})V({\bf{s}}),$$

in which *β* = 1/(*k*_B_*T*) is the inverse temperature, **s** is a set of CVs that are a function of the atomic coordinates **R**, the free energy is given within an immaterial constant by $F({\bf{s}})=-(1/\beta ){\mathrm{log}}\,\int\ d{\bf{R}}\ \delta [{\bf{s}}-{\bf{s}}({\bf{R}})]\ {e}^{-\beta {\mathrm{U}}({\bf{R}})}$, *U*(**R**) is the interparticle potential energy, and *p*(**s**) is a preassigned target distribution. The minimum of this convex functional is reached for

2 $$F({\bf{s}})=-V({\bf{s}})-\frac{1}{\beta }{\mathrm{log}}\,p({\bf{s}}),$$

which amounts to saying that in a system biased by *V*(**s**), the distribution is *p*(**s**).

In order to generate a multicanonical ensemble, one choose the potential energy *E* = *U*(**R**) as CV. Using VES we then determine the bias *V*(*E*) that leads to a flat distribution in the energy interval *E*_1_ < *E* < *E*_2_. This is obtained by choosing the target distribution

3 $$p(E)=\left\{\begin{array}{ll}1/({E}_{2}-{E}_{1}),&{\rm{if}}\,{E}_{1} \, < \, E \, < \, {E}_{2};\\ 0,&{\rm{otherwise}}.\end{array}\right.$$

By inserting in Eq. (1), the free energy as a function of *E* becomes

4 $$F(E)=V(E)-\frac{1}{\beta }{\mathrm{log}}\,p(E).$$

On the other hand, as *F*(*E*) can be written as

5 $$F(E)=E-\frac{1}{\beta }{\mathrm{log}}\,N(E).$$

one can reconstruct for the chosen interval the density of states *N*(*E*). From *N*(*E*), the thermal properties can be reconstructed using the fact that the partition function can be written as

6 $$Z=\int\ {e}^{-\beta E}N(E)dE.$$

If the *E* interval is appropriately chosen, one has in an interval *β*_1_ < *β* < *β*_2_

7 $$Z\approx \int_{{E}_{1}}^{{E}_{2}}{e}^{-\beta E}N(E)dE.$$

In ref. ^27^, a procedure is described such that once the desired *β*_1_ < *β* < *β*_2_ interval is chosen, the appropriate *E*_1_ < *E* < *E*_2_ interval in Eq. (3) is automatically chosen. Thus with a single simulation one can calculate the system properties in the entire *β*_1_ < *β* < *β*_2_ interval.

Extension to the multithermal–multibaric case is fairly straightforward. In addition to *E*, one shall also include in the CV set the volume *V* and perform a VES simulation with the following target distribution:

8 $$p(E,V)=\left\{\begin{array}{ll}1/{\Omega }_{E,V},&{\hbox{if there is at least one}}\,\,\beta ^{\prime} ,P^{\prime} \,\,{\hbox{such that}}\,\,\beta ^{\prime} {F}_{\beta ^{\prime} ,P^{\prime} (E,V)} \, < \, \epsilon \\ &{\rm{with}}\,{\beta }_{1} \, > \,\, \beta ^{\prime} \, > \, {\beta }_{2}\,{\rm{and}} \, {P}_{1}\, < \, P^{\prime} \, < \, {P}_{2}\hfill \\ 0,\hfill&{\rm{otherwise}}.\hfill\end{array}\right.$$

where Ω_*E*,*V*_ is a normalization constant, $\beta ^{\prime} {F}_{\beta ^{\prime} ,P^{\prime} (E,V)}$ is the free energy at temperature $T^{\prime} =1/({k}_{B}\beta ^{\prime} )$ and pressure $P^{\prime}$, and *ϵ* is a predefined energy threshold.

If we want to study first order phase transitions, a CV able to favor the transition between one phase and the other is needed. This leads to a VES problem with three CVs, E, V, and s. Once the run is converged, one can not only compute the properties of the system in a preassigned temperature *β*_1_–*β*_2_ and pressure *P*_1_–*P*_2_ region, but also draw the phase boundary. For more details, we refer to ref. ^28^.

### Collective variables

The explicit introduction of an appropriate CV is mandatory to drive the first-order phase transition. We adopt an orientationally targeted order parameters introduced recently by Piaggi and Parrinello^28^ building on the smooth overlap of atomic positions (SOAP) idea^33^. The starting point for the order parameter is the definition of the environment *χ* around an atom, and the associated local density *ρ*_*χ*_(**r**) is written as

9 $${\rho }_{\chi }({\bf{r}})=\sum_{i\in \chi }{e}^{-{\left|{{\bf{r}}}_{i}-{\bf{r}}\right|}^{2}/2{\sigma }^{2}},$$

where *i* runs over the neighbors in the environment *χ*, **r**_*i*_ are the coordinates of the neighbors relative to the central atom, and *σ*^2^ is the variance of the Gaussian functions. We then measure the difference between the environment *χ* and *χ*_0_ of the perfect crystal that contains *n* reference positions $\{{{\bf{r}}}_{j}^{0}\}(j=1\cdots n)$, where $\{{{\bf{r}}}_{j}^{0}\}$ are the crystallographic positions. The two environments are compared by performing the integral,

10 $${k}_{{\chi }_{0}}(\chi )=\int\ dr{\rho }_{\chi }({\bf{r}}){\rho }_{{\chi }_{0}}({\bf{r}}).$$

In the SOAP kernel^33^, a spherical average over all the possible orientations of the reference *χ*_0_ is performed. In the work of Piaggi and Parrinello^28^ the orientation of *χ*_0_ is instead kept fixed so as to facilitate the nucleation of defect free crystals. Once the rotational symmetry is removed, the integral in Eq. (10) is trivially performed leading to,

11 $${k}_{{\chi }_{0}}(\chi )=\sum_{i\in \chi }\sum_{j\in {\chi }_{0}}{\pi }^{3/2}{\sigma }^{3}{e}^{-{\left|{{\bf{r}}}_{i}-{{\bf{r}}}_{j}^{0}\right|}^{2}/4{\sigma }^{2}}.$$

The kernel function is then normalized

12 $${\tilde{k}}_{{\chi }_{0}}(\chi )=\frac{{k}_{{\chi }_{0}}(\chi )}{{k}_{{\chi }_{0}}({\chi }_{0})}=\frac{1}{n} \sum_{i\in \chi } \sum_{j\in {\chi }_{0}}{e}^{-| {{\bf{r}}}_{i}-{{\bf{r}}}_{j}^{0}{| }^{2}/4{\sigma }^{2}}.$$

For a system of *N* atoms, the kernel of each atom *i* can be denoted by ${\tilde{k}}_{{\chi }_{0}}({\chi }_{i})$ with *i* = 1, . . . , *N*. This quantity is a per atom crystallinity measure of the specific phase considered. For this reason, we use it as a fingerprint to follow the nucleation process. The CVs for the whole system is then constructed by counting the number of atoms that satisfy ${\tilde{k}}_{{\chi }_{0}}({\chi }_{i}) \, > \, {k}_{0}$ where *k*_0_ is chosen so as to separate the solid-like and liquid-like atoms. Finally, the CVs can be written in a continuous and differentiable fashion,

13 $$s=\mathop{\sum }_{i}^{N}\left(1-\frac{1-{\left({\tilde{k}}_{{\chi }_{0}}({\chi }_{i})/{k}_{0}\right)}^{p}}{1-{\left({\tilde{k}}_{{\chi }_{0}}({\chi }_{i})/{k}_{0}\right)}^{q}}\right),$$

where the parameters *p* and *q* control the steepness and the range of the switching function. In this way, *s* ≈ 0 for the liquid and *s* ≈ *N* for the solid structure.

This order parameter can be easily defined for any crystal structure by identifying the reference environments *χ*_0_. This is a straightforward calculation if the experimental solid phases that we are interested in are known. Furthermore, if the crystal structure of the solid phases is not known one could resort to crystal structure prediction algorithms^34^.

The kernel that defines the collective variables for *α*-Ga was defined using *σ* = 0.4 and the reference environment *χ*_0_ corresponded to the 13 nearest neighbors of the *α*-Ga lattice. The kernel for *β*-Ga was defined using *σ* = 0.35 and the reference environment *χ*_0_ corresponded to the 8 nearest neighbors of the *β*-Ga lattice. The kernel for Ga-II was defined using *σ* = 0.5 and the reference environment *χ*_0_ corresponded to the 12 nearest neighbors of the Ga-II lattice.

### NN representation of the potential energy surface

Among the many NN-based methods for constructing accurate force fields, we choose here the DeePMD method^21,23^. As in the work of Bonati-Parrinello^17^, our aim is to develop a potential whose validity is restricted to the physical phenomenon of interest namely the reconstruction of a portion of the Ga phase diagram and the nucleation of the relevant crystal phases. In the work of Bonati-Parrinello^17^ only *P* = 0 was explored and just one Si crystal structure needed to be considered. This facilitated the construction of the NN potential that was done in two steps. First, the nucleation was simulated using an effective classical potential and relevant configurations were extracted from this run. Second, the relative DFT energies and forces were calculated and on these results the NN potential was trained.

Our aims are more ambitious as we want to cover a broad-phase space region and the Ga iterations are far more subtle than that of Si case dealt within the Bonati-Parrinello work^17^. This requires a multi-step procedure. Again we start with a metadynamics calculation^25^ of *α*-Ga nucleation at ambient pressure using a classical potential^35^ and relevant configurations are extracted.

The DFT calculations are performed using the linear density approximations (LDA) exchange–correlation functional. Despite its simplicity, it has been shown that LDA is able to compare well with experiments^36^ and performs better than more complex functionals like the meta-GGA SCAN^37^. LDA has limitations, but nonetheless the overall agreement between simulations and experiment is remarkable. However, we note that the coexistence lines of the phase diagram (see Fig. 5) are shifted with respect to the experimental ones. This discrepancy could be owing to the accuracy of the exchange–correlation functional used. For instance, the estimated volume of *α*-Ga using LDA is ~18.97 Å^3^ per atom, slightly lower than that of experimental value 19.52 Å^3^. On the contrast, meta-GGA SCAN gives a value of 20.6 Å^3^. Such discrepancies caused by different functionals could result into different melting temperatures as shown in the literature^38,39^. Testing other functionals, or levels of theory is beyond the scope of our paper.

A first estimate of the potential NN1 is obtained by training a network on these configurations. The NN1 potential is then used in a number of isothermal–isobaric simulations of *α*-Ga, *β*-Ga, and Ga-II nucleation obtaining a new estimated NN2 potential. We then turned to multithermal–multibaric samplings and after two iterations we obtain a good potential able to describe bulk Ga in all the range of temperatures going from 150 K to 340 K and pressures from 1 bar to 2.6 GPa. The root mean square errors of the NN potential on the testing set are equal to 2.8 meV per atom for the energy and 60 meV per Å for the force.

The use of semiempirical potentials to generate the initial configurations does not limit the generality of our approach. The initial sampling could also have been done with a NN potential trained on ab initio MD simulations of the liquid and the solid phases. The empirical potential, if used, does not need to be particularly accurate, as all the required information of the configurations is then recalculated at the DFT level. Ga is a good example of this scenario since the empirical potential used here is quite poor and its description of the structure of the liquid phase is far from being adequate. Nevertheless, this did not prevent us from building a DFT quality potential energy surface by following the strategy depicted in Fig. 2.

### Nucleation energy barrier and nucleation rate calculations

In the CNT framework, the cost of forming a cluster of the new solid phase from liquid gallium can be expressed as ref. ^30^:

14 $$\Delta {G}^{CNT}=-\frac{4}{3}\pi {R}^{3}{\rho }_{s}\Delta \mu +4\pi {R}^{2}\gamma ,$$

in which R is the radius of the cluster, *ρ*_*s*_ is the number density of the solid phase, Δ*μ* is the chemical potential difference between the solid and the liquid phase, and *γ* is the interfacial free energy. The number of atoms in the cluster *n* can be estimated by $n=\frac{4}{3}\pi {R}^{3}{\rho }_{s}$. By inserting this result in Eq. (14) one obtains:

15 $$\Delta {G}^{CNT}(n)=-\Delta \mu n+\sigma {n}^{2/3},$$

where *σ* = ${(36\pi )}^{1/3}{\rho }_{s}^{-2/3}\gamma$. *ΔG*^*CNT*^ as a function of *n* exhibits a maximum located at,

16 $${N}_{c}=\frac{32\pi {\gamma }^{3}}{3{\rho }_{s}^{2}| \Delta \mu {| }^{3}}.$$

*N*_*c*_ is the number of atoms in the nucleus or critical cluster. By inserting *N*_*c*_ in Eq. (15) the nucleation energy barrier can be expressed as a function of Δ*μ* and *N*_*c*_ only,

17 $$\Delta G=\frac{1}{2}| \Delta \mu | {N}_{c}.$$

Furthermore, following the approach described in refs. ^40–42^, one can calculate the nucleation energy barrier *J* from the expression:

18 $$J={\rho }_{l}Z{f}^{+}\exp (-\Delta G/({k}_{B}T)),$$

in which (*ρ*_*f*_*Zf*^+^) is the kinetic prefactor, with *ρ*_*l*_ the number density of the liquid, *Z* the Zeldovich factor, and *f*^+^ the attachment rate of particles to the critical nucleus. The Zeldovich factor *Z* in the framework of CNT is

19 $$Z=\sqrt{| \Delta \mu | /(6\pi {k}_{B}T{N}_{c})},$$

in which *k*_*B*_ refers to the Boltzmann constant. The attachment rate *f*^+^ can be computed as a diffusion coefficient of the cluster size at which the cluster is critical^40–42^:

20 $${f}^{+}=\frac{\langle {\left(N(t)-{N}_{c}\right)}^{2}\rangle }{2t},$$

in which *N*(*t*) refers to the cluster size at simulation time *t*. The attachment rates *f*^+^ of *α*-Ga at 170 K and *β*-Ga at 180 K are estimated to be 4.7e^14^ and 3.8e^15^ s^−1^, respectively.

### MD setup

MD simulations were performed using LAMMPS^43^ and a development version of PLUMED 2^44^ supplemented by the VES module^45^. The temperature was controlled using the stochastic velocity rescaling thermostat^46^ and the pressure was kept constant employing the isotropic version of the Parrinello–Rahman barostat^47^. The integration time step used is 2 fs and the relaxation times for the thermostat and the barostat were set to 0.1 and 1 ps, respectively.

For the multithermal–multibaric simulations, a bias potential was constructed using VES and employing the energy *E*, the volume *V* and *s* as CVs. Legendre polynomials of order 8 were used in each dimension for a total of 729 variational coefficients. The integrals of the target distribution were performed on a grid of size 40 × 40 × 40. Four multiple walkers were used for the simulations. For *α*-Ga, the intervals where the polynomials were defined are  −28179800 < *E* < −28178000 kJ/mol, 2.54 < *V* < 2.80 nm^3^, and 0 < *s* < 144. The target distribution was smoothed using Gaussians with *σ*_*E*_ = 200 kJ/mol, *σ*_*V*_ = 0.05 nm^3^, and *σ*_*s*_ = 2. The exploration threshold was set to *ϵ* = 200 *k*_*B*_*T*. The coefficients of the bias potential were optimized every 500 steps using the averaged stochastic gradient descent algorithm with a step size of *μ* =  5 kJ/mol. For *β*-Ga, the parameters used were the following: −28179400 < *E* < −28178100 kJ/mol, 2.48 < *V* < 2.76 nm^3^, 0 < *s* < 144, *σ*_*E*_ = 100 kJ/mol, *σ*_*V*_ = 0.05 nm^3^, *σ*_*s*_ = 2, *ϵ* = 40 *k*_*B*_*T* and *μ* = 1 kJ/mol. For Ga-II the parameter were:  −28179300 < *E* < − 28178300 kJ/mol, 2.42 < *V* < 2.70 nm^3^, 0 < *s* < 144, *σ*_*E*_ = 100 kJ/mol, *σ*_*V*_ = 0.05 nm^3^, *σ*_*s*_ = 2, *ϵ* = 40 *k*_*B*_*T*, and *μ* = 1 kJ/mol.

We have used the well-tempered variant of metadynamics (WTMetaD)^26^ with adaptive Gaussians^48^ to carry out nucleation simulations. The bias factor and initial Gaussian height for *α*-Ga and *β*-Ga in the system with 144 atoms were set to be 100 and 50 kJ/mol, and 80 and 20 kJ/mol, respectively. The upper and lower limit of the Gaussian width were set to 2 and 0.2. For the system with 2560 atoms to simulate *α*-Ga nucleation, a bias factor of 320 and initial Gaussian height 300 kJ/mol were used, whereas for the system with 2400 atoms to simulate *β*-Ga nucleation, they were set to be 200 and 200 kJ/mol, respectively. The upper and lower limit of the Gaussian width were equal to 1 and 30. The reweighted FES shown in Fig. 7 as a function of the fraction of solid-like atoms *f*_*s*_ and fraction of Ga_2_ dimers *f*_*d*_ for the nucleation of *α*-Ga and *β*-Ga were calculated following the reweighting procedure given in ref. ^49^.

In the seeding MD simulations, five initial configurations were extracted from the nucleation trajectories of *α* with 2560 atoms in the system and *β*-Ga with 2400 atoms obtained by the WTMetaD simulations. After 0.2 ns equilibrium simulation, the sizes of the cluster in the chosen configurations in the case of *α*-Ga were about 130, 181, 238, 342, and 505 atoms, whereas in the case of *β*-Ga, they were ~216, 310, 402, 490, and 520 atoms.

As the crystal might form in directions that are not aligned with the simulation box during the MD simulation, we introduced the following quantity to avoid such undesired phenomenon as in ref. ^28^,

21 $${s}_{c}=\frac{{Q}_{6}-{Q}_{6}^{l}}{{Q}_{6}^{s}-{Q}_{6}^{l}}-\frac{s-{s}^{l}}{{s}^{s}-{s}^{l}},$$

where *Q*_6_ is the global Steinhardt parameter as defined in refs. ^50,51^ and *s* is the collective variable that induces the nucleation. As discussed in ref. ^28^, the rationale behind *s*_*c*_ is that *s*_*c*_ is close to zero only if *Q*_6_ and *s* increase simultaneously. By keeping *s*_*c*_ close to zero, crystals with orientations different from the desired one are avoided. To achieve this, a restraining potential is applied:

22 $$V({s}_{c})=\left\{\begin{array}{ll}k{\left({s}_{c}-{s}_{c}^{0}\right)}^{2},&{\rm{if}}\ {s}_{c} \, > \, {s}_{c}^{0};\\ 0,&{\rm{otherwise}},\end{array}\right.$$

in which *k* = 10^5^ kJ/mol. For *α*-Ga, *β*-Ga, and Ga-II, ${s}_{c}^{0}$ equal to 0.25, 0.14, and 0.18, respectively. The distance cutoff in the calculation of *Q*_6_ was 0.35 nm.

### DFT calculations setup

DFT^52^ calculations are performed using the Quickstep module^53^ of the CP2K program^54^. The 3*d*^10^3*s*^2^*p*^1^ electrons were treated explicitly, using LDA as exchange–correlation functional. The single-point energy and forces calculations for the training set used a energy cutoff of 600 Ry. The threshold for energy convergence is set to 10^−11^ and the one related to SCF cycles is set to 10^−6^. A Fermi Dirac smearing with 0.025 eV of the occupation number at the Fermi level is used, and mixing of the electronic density in *k*-space is also used. There were 144 atoms in the study of liquid-*α* and liquid Ga-II phase transitions, where 160 atoms were used in the case of *β*-Ga. A *k*-points grid of 2 × 2 ×  2 is adopted.

### DeePMD setup

The DeePMD-kit package^23^ is used for the training of the NN potential and to interface the NN potential to LAMMPS. A NN with five hidden layers is used, with a number of neurons per layer equal to (240,120,60,30,10). The total number of training configurations is 28,000. The network is trained with the ADAM optimizer, with an exponentially decaying learning rate from 1e-3 to 1e-5. The prefactors of the energy, the force and the virial terms in the loss functions change during the optimization process from 0.2 to 1, from 500 to 1, and from 0.02 to 0.2.

## Supplementary information

Supplementary Information

## Data Availability

All the data and the input files necessary to reproduce the results contained in this paper are available in the Materials Cloud repository via https://archive.materialscloud.org/2020.0039/v1.

## Code availability

LAMMPS, Plumed, and DeepMD are free and open source codes available at https://lammps.sandia.gov, https://www.plumed.org, and http://www.deepmd.org, respectively.
