# Supplementary material for: Ab initio phase diagram and nucleation of gallium
Source: Nat Commun. 2020 May 27;11:2654. doi: 10.1038/s41467-020-16372-9 (PMC7253470; doi:10.1038/s41467-020-16372-9)
Supplement: Supplementary file 1 — Supplementary Information [file 41467_2020_16372_MOESM1_ESM.pdf]

Supplementary Information for
"$Ab - initio$ phase diagram and nucleation of gallium"

Haiyang Niu[1,2,3], Luigi Bonati[4,3], Pablo M. Piaggi[2,3], and Michele Parrinello[2,3,5]
[1] *State Key Laboratory of Solidification Processing,*
International Center for Materials Discovery, School of Materials Science and Engineering,
Northwestern Polytechnicial University, Xi'an 710072, P. R. China
[2] *Department of Chemistry and Applied Biosciences,*
ETH Zurich c/o USI Campus, Via Giuseppe Buffi 13, 6900 Lugano, Switzerland
[3] *Facoltà di Informatica, Instituto di Scienze Computationali,*
and National Center for Computational Design and Discovery of Novel Materials MARVEL,
Università della Svizzera italiana (USI), Via Giuseppe Buffi 13, 6900 Lugano, Switzerland
[4]*Department of Physics, ETH Zurich, c/o Universit'a della Svizzera italiana,*
Via Giuseppe Buffi 13, CH-6900, Lugano, Switzerland and
[5] *Istituto Italiano di Tecnologia, Via Morego 30, 16163 Genova, Italy*
(Dated: March 29, 2020)

FREE ENERGY DIFFERENCES CALCULATION

We follow the procedure discussed in Ref. [1] to calculate the free energy differences. In order to keep the paper self-consistent, we reproduce here this part as discussed in details in Ref. [1]. In order to calculate free energy differences between the liquid and the solid $\Delta G(T,P)$ at temperature T and pressure P we make use of the collective variable s which is a function of the atoms' coordinates $\mathbf{R}$. If $s < s_0$ the configuration of the system is compatible with the liquid state and if $s > s_0$ it is compatible with the solid state. Using s the free energy difference can be expressed as

$$\Delta G(T,P) = -\frac{1}{\beta}\log\left(\frac{\mathcal{P}_{T,P}(s>s_0)}{\mathcal{P}_{T,P}(s<s_0)}\right), \tag{1}$$

where $\beta = 1/k_BT$ is the inverse temperature, k_B is the Boltzmann constant, and $\mathcal{P}_{T,P}(s>s_0)$ and $\mathcal{P}_{T,P}(s<s_0)$ are the probabilities of finding the system in the solid and liquid state, respectively. In the isothermal-isobaric ensemble at temperature T and pressure P the probability of finding a configuration $\mathbf{R}$ is $e^{-\beta(U(\mathbf{R})+P\mathcal{V})}/Z_{\beta,P}$ where $Z_{\beta,P}$ is the appropriate partition function and thus Eq. (1) can be rewritten as

$$\Delta G(T,P) = -\frac{1}{\beta}\log\left(\frac{\int\limits_{s>s_0} ds \int d\mathbf{R} \int d\mathcal{V}\, e^{-\beta(U(\mathbf{R})+P\mathcal{V})}\delta(s-s(\mathbf{R}))}{\int\limits_{s<s_0} ds \int d\mathbf{R} \int d\mathcal{V}\, e^{-\beta(U(\mathbf{R})+P\mathcal{V})}\delta(s-s(\mathbf{R}))}\right), \tag{2}$$

where $U(\mathbf{R})$ is the potential energy and $\mathcal{V}$ is the volume. Therefore our goal is to calculate the ratio of the integrals in Eq. (2) in a region of the temperature-pressure plane.

Once the bias potential has converged, the mean value of an observable in the isothermal-isobaric ensemble at temperature T' and pressure P' can be calculated from the multithermal-multibaric simulation using,

$$\langle O(\mathbf{R},\mathcal{V})\rangle_{T',P'} = \frac{\langle O(\mathbf{R},\mathcal{V})w(\mathbf{R},\mathcal{V})\rangle_{T,P,V}}{\langle w(\mathbf{R},\mathcal{V})\rangle_{T,P,V}}, \tag{3}$$

where $w(\mathbf{R},\mathcal{V}) = e^{(\beta-\beta')E(\mathbf{R})+(\beta P-\beta' P')\mathcal{V}}e^{\beta V}$, $\langle\cdot\rangle_{T',P'}$ is the ensemble average in the isothermal-isobaric ensemble at temperature T' and pressure P', and $\langle\cdot\rangle_{T,P,V}$ is the ensemble average at temperature T and pressure P with bias potential V.

Once the distribution $p(E,\mathcal{V},s)$ has been determined, statistics can be gathered to calculate free energy differences $\Delta G(T',P')$ using Eq. (2) for $T_1 < T' < T_2$ and $P_1 < P' < P_2$. Since a biased ensemble is being sampled, Eq. (3) must be used to calculate $\Delta G(T',P')$. In order to use Eq. (3), we recast Eq. (2) as an ensemble average,

$$\Delta G(T',P') = -\frac{1}{\beta}\log\left(\frac{\langle H(s-s_0)\rangle_{T',P'}}{\langle 1-H(s-s_0)\rangle_{T',P'}}\right) \tag{4}$$

where

$$H(s-s_0) = \begin{cases} 1 \text{ if } s > s_0 \\ 0 \text{ if } s < s_0 \end{cases} \tag{5}$$

is the Heaviside function and s_0 is the value of the order parameter that defines the watershed between the liquid and the solid. We have chosen $s_0 = N/2$, that is to say, all configurations in which less than half the atoms are solid-like are considered liquid and those with more than half solid-like atoms are classified as solid. This choice is not crucial since the regions of s that contribute most to $\Delta G(T',P')$ are $s \approx 0$ and $s \approx N$. Now that $\Delta G(T',P')$ is a function of ensemble averages it is easy to employ Eq. (3) to calculate it.

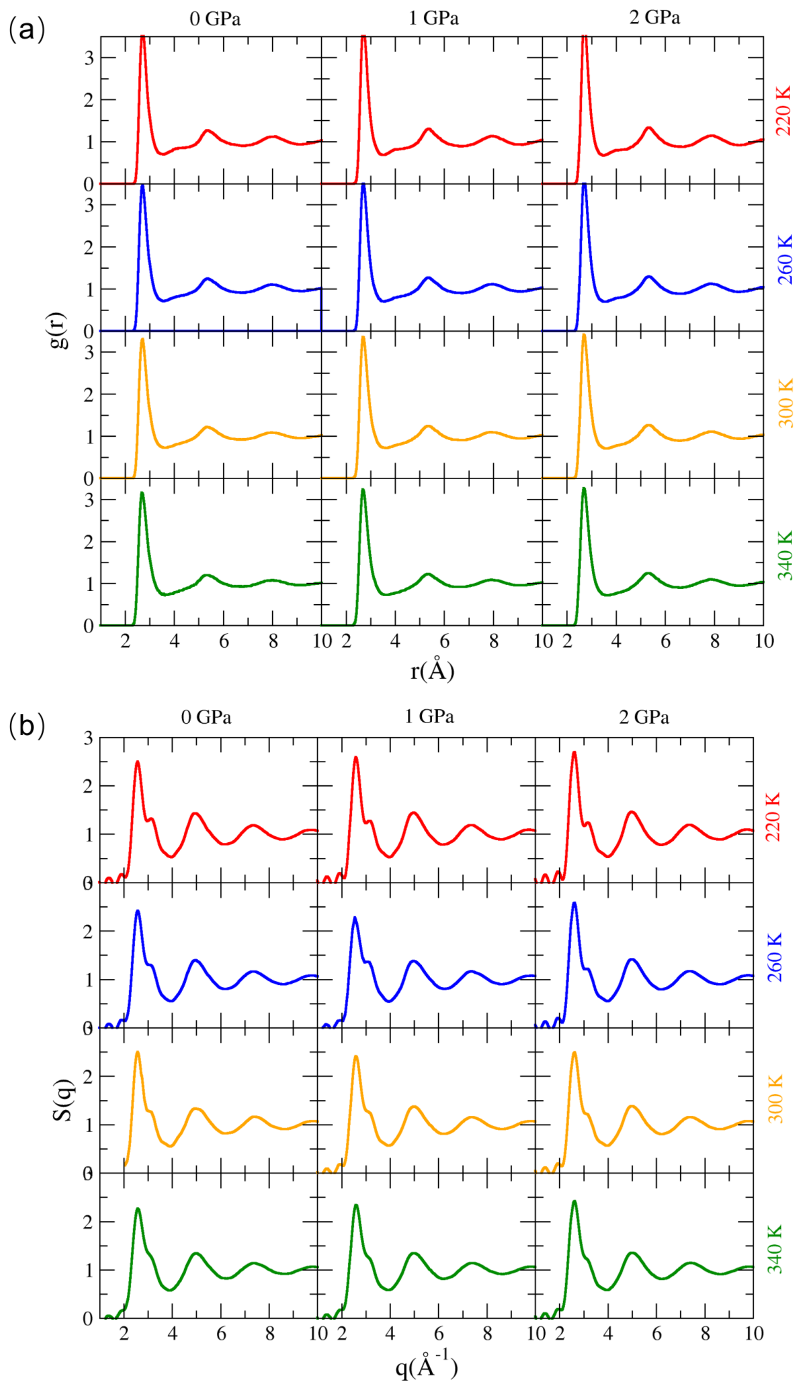

Supplementary Figure 1. Structure properties of liquid gallium at different temperatures and pressures. a) Radial distribution functions $g(r)$ and b) static structure factors $S(q)$ of liquid gallium are shown in red, blue, orange and green lines for the temperatures of 200, 260, 300, and 340 K, respectively. The columns correspond to pressures of 0, 1 and 2 GPa.

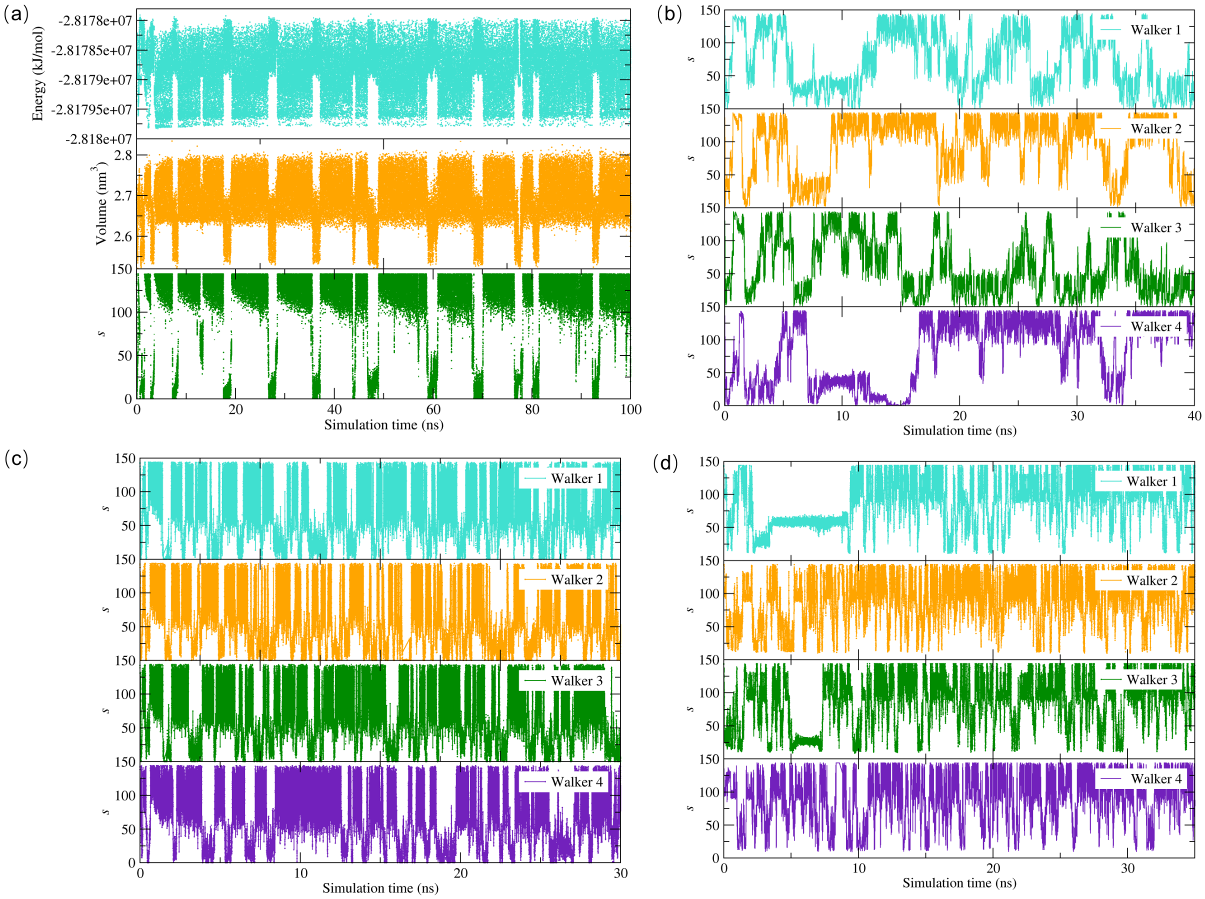

Supplementary Figure 2. Collective variables as a function of simulation time in the multithermal-multibaric simulations. a) Energy E, volume V and order parameter s vs. time for the simulation of liquid-α Ga transition with one walker. Order parameter s vs. time for the simulations of b) liquid-α Ga, c) liquid-β Ga, and d) liquid-Ga II transitions with four walkers.

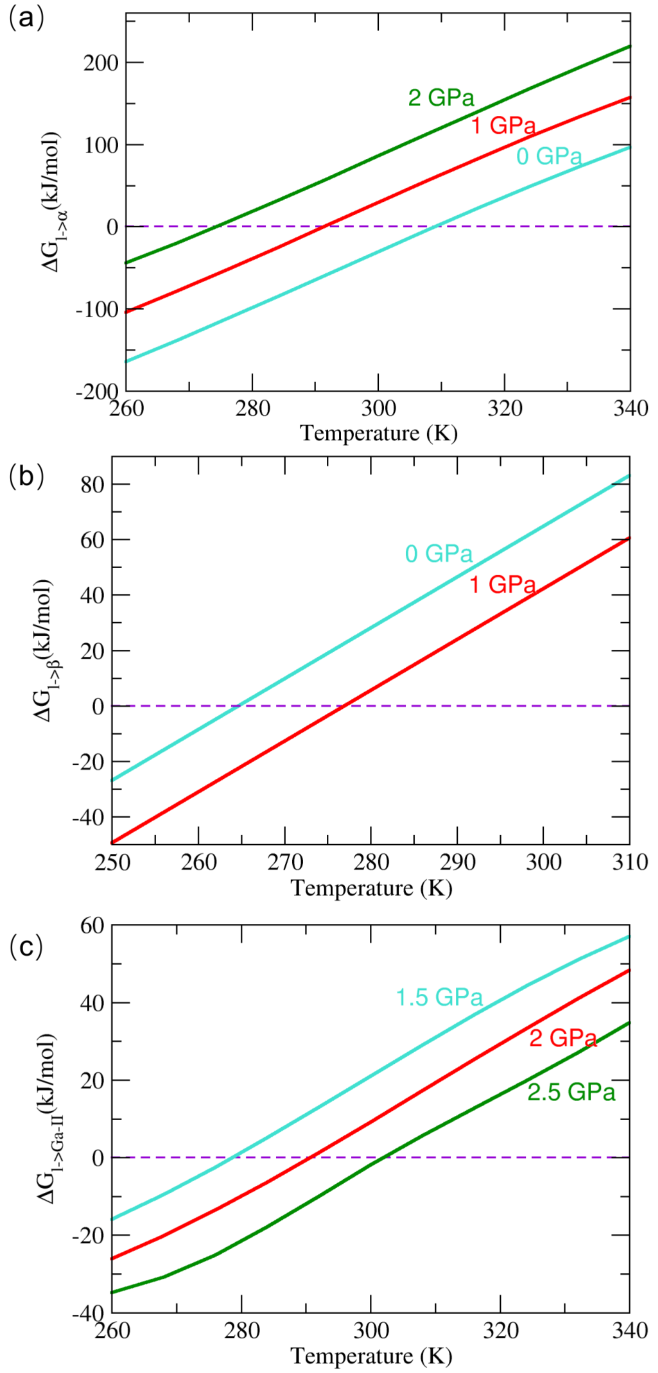

Supplementary Figure 3. Free energy differences ΔG between the liquid and a) α-Ga, b) β-Ga, and c) Ga-II phases at different pressures.

[1] P. M. Piaggi and M. Parrinello, The Journal of chemical physics **150**, 244119 (2019).
